# Potential Pro-Tumorigenic Effect of Bisphenol A in Breast Cancer via Altering the Tumor Microenvironment

**DOI:** 10.3390/cancers14123021

**Published:** 2022-06-19

**Authors:** Youngjoo Kwon

**Affiliations:** Department of Food Science and Biotechnology, Ewha Womans University, 52 Ewhayeodae-gil, Seodaemun-gu, Seoul 03760, Korea; youngjoo.kwon@ewha.ac.kr

**Keywords:** adipocytes, bisphenol A, breast cancer, extracellular matrices, endocrine disruptors, fibroblasts, immune cells, tumor microenvironment

## Abstract

**Simple Summary:**

Bisphenol A (BPA) is primarily used to produce polycarbonate plastics, such as water bottles. Exposure to BPA has been shown to increase the growth of breast cancer cells that depend on estrogen for growth due to its ability to mimic estrogen. More recent studies have suggested that BPA also affects the cellular and non-cellular components that compose tumor microenvironments (TMEs), namely the environment around a tumor, thereby potentially promoting breast cancer growth via altering the TME. The TME plays an essential role in cancer development and promotion. Therefore, it is crucial to understand the effect of BPA on breast TMEs to assess its role in the risk of breast cancer adequately. This review examines the potential effects of BPA on immune cells, fibroblasts, extracellular matrices, and adipocytes to highlight their roles in mediating the carcinogenic effect of BPA, and thereby proposes considerations for the risk assessment of BPA exposure.

**Abstract:**

BPA, a chemical used in the preparation of polycarbonate plastics, is an endocrine disruptor. Exposure to BPA has been suggested to be a risk factor for breast cancer because of its potential to induce estrogen receptor signaling in breast cancer cells. More recently, it has been recognized that BPA also binds to the G protein-coupled estrogen receptor and other nuclear receptors, in addition to estrogen receptors, and acts on immune cells, adipocytes, and fibroblasts, potentially modulating the TME. The TME significantly impacts the behavior of cancer cells. Therefore, understanding how BPA affects stromal components in breast cancer is imperative to adequately assess the association between exposure to BPA and the risk of breast cancer. This review examines the effects of BPA on stromal components of tumors to highlight their potential role in the carcinogenic effect of BPA. As a result, I propose considerations for the risk assessment of BPA exposure and studies needed to improve understanding of the TME-mediated, breast cancer-promoting effect of BPA.

## 1. Introduction

According to the GLOBOCAN 2020 estimates on cancer incidence and mortality produced by the International Agency for Cancer, breast cancer is the most commonly diagnosed cancer in women, accounting for 24.5% of cancer incidence and 15.5% of cancer deaths [1]. Breast cancer ranks first in incidence in women in most countries [1]. Historically, Asian women have a lower incidence of breast cancer than women in North America and Western Europe. However, incidence rates in Asia have continued to increase rapidly during the past few decades relative to those in Western countries, where incidence rates increased until the mid-to-late 1990s and then plateaued [2,3].

Compared to women in Western countries, there is also a trend of an earlier onset of breast cancer in women from many parts of Asia; in South Korea and Thailand, the median age at diagnosis is 49 to 50 years [3]. The cause of this difference is unclear, but it has been suggested that the cohort effect contributes to the younger age of onset in the Asian population; there was no difference in the age-specific rates between the two populations when rates were measured as longitudinal (early vs. late cohorts) [4,5,6,7]. This cohort effect in the Asian population may indicate the prevalence of risk factors for breast cancers in Western countries (e.g., early menarche, late menopause, delayed childbearing, fewer children, and obesity) in succeeding generations of Asian women [4]. Additionally, the introduction of screening programs, such as mammography, in Asia may have contributed to the increased incidence in later generations [3,8]. Consistent with this view, the median age of diagnosis has increased over time in Asian countries, which was apparent in some countries, such as Japan [8].

In addition to the increased lifetime exposure to estrogens, the rapid increase in breast cancer incidence in Asian countries may be related to high exposure to endocrine-disrupting chemicals (EDCs). During the fast industrialization and economic growth in many Asian countries, the production and use of plastics rapidly increased, which may have led to abrupt exposure to plastic-derived contaminants, such as bisphenols and phthalates [9]. Bisphenol A (BPA) has been used to produce epoxy resins and polycarbonate plastics since the 1950s [10,11]. Epoxy resins coat the inside of metal products, such as food cans and water pipes [12]. Polycarbonate plastics are often used in containers that store food and beverages, such as water bottles [10]. BPA is also used as a color developer to make the thermal paper on which most receipts are printed [13]. BPA is considered safe for its intended use because its estimated human exposure levels do not exceed the derived “safe” levels, the tolerable daily intake (TDI) [12]. However, using BPA to produce baby feeding bottles has been banned in the United States (US) and the European Union (EU) due to uncertainty about the effect of BPA exposure in infants [14]. In line with this, the European Food Safety Authority (EFSA) reduced the TDI of BPA from 50 to 4 μg/kg body weight (bw)/day in 2015, and the EU included BPA on the list of candidate substances of serious concern in 2017, which is the first step toward restricting the importation and use of a chemical [13]. Thus, health concerns regarding BPA exposure remain. Meanwhile, the global BPA market has increased, and annual production of BPA is projected to reach six million tonnes by 2023 [15], suggesting that BPA is extensively used, and thus, daily exposure to BPA is unavoidable. For adults in the US, the overall dietary intake was estimated to be 12.6 ng/kg bw/day, and the total BPA intake estimated from urine measurements via the National Health and Nutrition Examination Survey is between 30 to 70 ng/kg bw/day [16]. Given the continued exposure and bioaccumulation of BPA, long-term exposure to BPA can significantly impact human health outcomes [17].

Some epidemiologic studies have failed to demonstrate the association between exposure to EDCs and breast cancer incidence, probably due to the difficulty of assessing exposure [18]. However, the risk of breast cancer is strongly related to occupational exposure to EDCs, such as food canning [19]. Premenopausal women working for 10 years in food canning showed a five-fold increase in breast cancer incidence [19]. Additionally, BPA levels were higher in both the urine and the adipose tissues of breast cancer patients than in women who underwent mammoplasty [20]. EDCs disrupt hormonal homeostasis by mimicking or inhibiting the binding of a hormone to its receptor. Notably, the ability of BPA to mimic estrogen and exert weak estrogenic activities has been speculated to be the mechanism that increases the risk of estrogen-dependent breast cancers [20]. In contrast, the hazardous effect of BPA has been suggested in other cancers that may not be related to the activation of estrogen receptors (ERs), such as triple-negative breast cancer and liver cancer [21,22]. In this regard, the literature shows that as an active endocrine substance, BPA can also interact with the androgen receptor and modulate the activities of other nuclear receptors, such as estrogen-related receptor gamma (ERR-γ), aryl hydrocarbon receptor (AhR), and peroxisome proliferator-activated receptor gamma (PPAR-γ), as well as toll-like receptors (TLRs) [23,24,25]. Furthermore, the transmembrane G protein-coupled estrogen receptor (GPER) may be a target for a broad spectrum of EDCs, including BPA [26,27]. GPER binds estrogen with high affinity and mediates rapid nongenomic signaling events, resulting in cancer cell proliferation [28]. Therefore, BPA exposure may increase the risk of breast cancer via multiple signaling pathways.

Notably, low oral doses of BPA significantly decreased tumor latency and increased tumor multiplicity, tumor volume, and the incidence of metastasis in mammary tumor virus (MMTV)-*ErbB2/neu* transgenic mice [29]. Previous studies investigating the association of BPA exposure with the risk of breast cancer largely focused how BPA affected cancer cells [30]. However, cancer progression is greatly influenced by the surrounding tumor microenvironment (TME) that has cellular components (fibroblasts, adipocytes, immune cells) and a non-cellular component, the extracellular matrix (ECM) [31,32,33]. Furthermore, fibroblast, adipocyte, and immune cells express ERs, GPER, or both, implying a possible role of BPA in cancer progression via altering signaling mediated by ERs or GPER [28,34,35]. It follows that BPA exposure may lead to stromal alterations that can promote cancer initiation or progression, so understanding how BPA affects the stromal component of breast tumors is imperative to adequately assess how BPA exposure increases the risk of breast cancer. In this review, I discuss the effects of BPA on the stromal component of tumors to highlight the potential roles of immune cells, adipocytes, fibroblasts, and ECMs that compose the TME in the carcinogenic effect of BPA, and thereby propose that the effect of BPA on the stromal component should be considered when assessing the risk of BPA exposure.

## 2. Immune Cells

The immune system is divided into innate immunity, which delivers rapid and non-specific responses, and adaptive immunity, which engages antigen-dependent and antigen-specific mechanisms [36]. Adaptive immune responses are mediated by B lymphocytes (B cells) and T lymphocytes (T cells). During antigen-dependent activation, premature T cells can mature into memory cells or effector cells. Effector cells are CD8+ cytotoxic T lymphocytes (CTLs) that detect and destroy virus-infected or cancer cells and CD4+ T helper (Th) lymphocytes that release specific cytokines to activate other immune cells (e.g., B cells and CTLs) [37]. Similarly, upon activation by an antigen, B cells can develop into memory cells or antibody-secreting effector cells that release antibodies to neutralize or destroy the target antigen [38].

Innate immune cells include macrophages, neutrophils, eosinophils, basophils, mast cells, and dendritic cells (DCs). These cells recognize pathogen-associated molecular patterns (PAMPs), common repetitive molecular structures associated with microorganisms, or damage-associated molecular patterns (DAMPs) released from tissue injuries or cancer cells [36]. The binding of PAMPs or DAMPs to TLRs or other pattern recognition receptors activates innate immune cells that initiate immune responses that can directly contribute to tissue inflammation or immune resolution by phagocytosis and the secretion of immune-modulating mediators (e.g., cytokines, chemokines, and proteases) [39]. Alternatively, the cellular immune reaction can be indirectly achieved via professional antigen-presenting cell (APC)-mediated adaptive immune responses [36]. APCs (e.g., DCs and macrophages) display foreign antigens (including tumor antigens) on their surface and migrate to lymphoid organs, where APCs present the antigens to adaptive immune cells, which can modulate the activation, differentiation, and effector functions of adaptive immune cells, thereby linking innate and adaptive immune responses [37,40].

Distinct types of immune cells perform diverse roles within the TME. In principle, immune cells can protect the body from developing cancer by killing cancer cells; however, they can also shape cancer immunogenicity to avoid immune recognition [33], support the growth of cancer cells, and promote invasion and metastasis [12]. Therefore, immune cells have a dynamic relationship with cancer cells, a relationship that can both support and inhibit cancer development and progression. BPA, as an immune disruptor is less studied than BPA as an endocrine disruptor [41]. However, BPA has been shown to modulate immune function through various receptors. Most immune cells express ERs, which significantly affect both innate and adaptive immune responses [42,43]. Additionally, GPER is expressed in a subset of immune cells, including B cells, T cells, neutrophils, and macrophages [44]. Furthermore, the immune response can be modulated by the binding of BPA to nuclear receptors other than ERs—such as ERRs, AhR, PPARs, and TLRs [45]. Altered immune function upon exposure to BPA may affect cancer growth and progression by forming a cancer-promoting TME.

### 2.1. Immunotoxicity

T cells that specifically recognize cancer can trigger an adaptive immune response, which is critical in fighting cancer cells. In particular, the CTL function has been extensively explored as a cancer therapy. For an effective adaptive immune response, the proliferation of antigen-specific T cells is fundamental because their competence depends entirely on cell renewal and clonal expansion [46]. As such, telomere intactness is critical in preserving immune functions [47,48]. Short telomeres of human peripheral blood mononuclear cells (PBMCs) have been implicated in many immune-related diseases and cancers, regardless of the malignancies of immune cell lineages (e.g., leukemia) or other cell types (e.g., lung cancer) [49,50].

BPA exposure may suppress T cell function by altering telomere length or the telomerase responsible for maintaining the telomere length through the addition of guanine-rich repetitive sequences [51]. High BPA exposure is associated with shorter relative telomere length in peripheral blood; women with a higher urinary BPA concentration (≥2.44 μg/g creatinine) had significantly shorter relative telomere length than women with a lower urinary BPA concentration (<1.26 μg/g creatinine) [52]. BPA treatment also reduced the proliferation of human PBMCs induced by phytohemagglutinin (PHA), although the BPA treatment conditions (50–200 μM for 48 h) of that study may not be relevant to human applications [53]. More notably, long-term (22 days) low-dose BPA (1–3 nM) treatment of human PBMCs significantly reduced telomerase activity upon activation of T cells using anti-CD3 and anti-CD28 antibodies [54]. In the same study, the effect of BPA on telomerase was inhibited by co-treatment with inhibitors of ERs (1,3-bis(4-hydroxyphenyl)-4-methyl-5-[4-(2-piperidinylethoxy)phenol]-1H-pyrazole dihydrochloride or MPP and 4-[2-phenyl-5,7-bis(trifluoromethyl)pyrazolo[1,5-*a*]pyrimidin-3-yl]phenol or PHTPP), GPER (G15), and extracellular signal-regulated kinase 1/2 or ERK1/2 (U0126), suggesting these proteins are involved in BPA-mediated telomerase inhibition. Long-term treatment with low-dose BPA also increased DNA-damage frequency and decreased the proliferation rate of PBMCs [54]. In a successive study, the immunotoxic effect of BPA was shown to be largely specific to CTLs [55]. Long-term low-dose (0.3 and 3 nM) treatments of BPA inhibited proliferation, and reduced telomere length, mitochondrial DNA copy number, and the protein expression of human telomerase reverse transcriptase (hTERT), a catalytic component of the telomerase complex protein, in CTLs but not in Th cells isolated from healthy individuals in response to anti-CD3/CD28 antibodies [55]. These lines of evidence suggest that long-term exposure to non-occupational concentrations of BPA (10 nM range) can decrease telomere length in a subset of human T cells, which may decrease their capability to expand upon activation.

By contrast, a comprehensive evaluation of the immune response in splenocytes isolated from BPA-treated rats concluded that the BPA-mediated changes were moderate and not persistent over the long term. Thus, BPA exposure is unlikely to compromise immune competence in adult rats [56]. In the aforementioned study, the ability of splenocytes to induce immunoglobulin production and activation of various immune cells was not significantly altered by exposure to a wide range of BPA doses (2.5–2500 μg/kg bw/day) for up to 1 year. However, it is noteworthy that low-dose (2.5 μg/kg bw/day) BPA given over a year reduced the proliferation of isolated splenocytes upon activation with anti-CD3 and anti-CD28 antibodies in female rats, despite an overall increase in proliferation in response to lipopolysaccharide (LPS) or pokeweed mitogen [56]. Anti-CD3/CD28 treatment activates all classes of lymphocytes, either directly (T cells) or indirectly (B and natural killer [NK] cells), whereas LPS specifically targets monocytes [57]. Another animal study found that pretreating female BALB/c mice with BPA (5 mg/kg bw/day) for 5 days decreased T cells in the spleen compared to the untreated control [58], although a limitation of the study was the use of a high dose of BPA delivered via subcutaneous injection [58]. Notably, the effect of BPA continued in CTLs even after the cessation of the treatment [58]. This suggests that T cells may be more susceptible to BPA-induced toxicity than other immune cells (Table 1). BPA-exposed individuals may be more susceptible to developing cancer because CTL function is essential to prevent cancer development.

Studies have also suggested that BPA exposure may modulate the innate immune function responsible for early defense [58]. Treatment with BPA modulated the surface expression of CD11c, CD14, CD15, CD16, and CD62L, which are known to participate in the basic functions related to the recognition and elimination of pathogens in neutrophils [72]. In particular, CD16 expression on neutrophils isolated from healthy men and women decreased after BPA treatment over a wide range of concentrations from 16 nM to 12 μM [72], which suggests dysfunction in phagocytosis [73]. Additionally, the elimination rate of bacteria was reduced at the infection site in BPA-treated mice within 24 h (early-stage infection) after challenging the mice with the non-pathogenic bacteria *Escherichia coli* K-12 [58]. Consistently, neutrophils obtained from mice treated with BPA (5 mg/kg bw/day) had less phagocytosis and a lower ability to kill bacteria than the neutrophils derived from vehicle-treated mice [58]. Although limited studies suggest that BPA exposure may also affect neutrophil function, more studies will be required in the context of cancer.

### 2.2. Alteration of Immune Cell Infiltration and Polarization

Cancer-associated immune responses can both suppress and facilitate the progression of cancer. This contrary effect may largely depend on the cell type and the polarization of infiltrated immune cells. In addition to CTLs, various immune cells (e.g., macrophages, NK cells, neutrophils, and DCs) can infiltrate a tumor to various degrees. The tumor-infiltrating immune cell is related to the disease outcome of various cancers, including breast cancer [74,75]. For example, Th1 cells, CTLs, NK cells, M1 macrophages, and DCs protect against tumor growth, whereas Th2 or regulatory T cells (Tregs) and M2 macrophages promote tumor growth [76].

The distinct roles of Th1 and Th2 cells and M1 and M2 macrophages in tumor development and progression have been widely recognized, while different subpopulations of neutrophils (N1 and N2) and their roles in cancer have been more recently appreciated [77]. The M1 macrophage is associated with a high level of phagocytosis and the secretion of pro-inflammatory cytokines, which contribute to cancer cell elimination [78]. Similarly, Th1 cells secrete a high level of interferon gamma (IFN-γ) that stimulates the immune response to protect against cancer cells. In contrast to type I immunity characterized by Th1 cells and M1 macrophages, immunosuppressive immune cell types produce high levels of immunosuppressive cytokines, besides other immunosuppressive molecules, and shift toward a type II immune response [79,80,81]. Type II immunity is associated with polarization into Th2 cells and M2 macrophages, suppressing the anti-tumor immune responses of CTLs, Th1 cells, and M1 macrophages [80,81].

The human breast TME displays Th2-mediated type II immunity features that promote cancer progression [82]. Studies have shown the importance of Th cells in mediating pro- or anti-tumorigenic effects. For example, C-C motif chemokine ligand (CCL) 5 exerted its pro-tumorigenic effects via induction of Th2 polarization, promoting the metastasis of breast cancer cells [83]. Conversely, increasing Th1 polarization reduced the growth of breast tumors [84]. Macrophages (tumor-associated macrophage, TAM) constitute a significant component of the TME. A high fraction of M2 TAMs was observed in human breast tumors, which was related to poor outcomes [85,86]. Animal models and in vitro culture experiments revealed that M2 polarization stimulates the proliferation of cancer cells, mediates immunosuppression, and induces angiogenesis in breast cancer [79]. Exposure to BPA may alter the polarization and infiltration of Th cells or macrophages, as described below (Section 2.2.1 and Section 2.2.2), thereby producing a pro-tumorigenic TME.

#### 2.2.1. Polarization of Th Cells

After activation, mature naïve forms of Th cells (Th0) can differentiate into distinct functional effector cells, including Th1, Th2, Th17, and Tregs. Each effector cell type is activated by a specific set of cytokines and transcription factors [87,88]. For example, IFN-γ drives Th1 cell polarization, whereas interleukin (IL)-4 and IL-10 inhibit Th1 cell polarization [89]. Conversely, IL-4 drives but IFN-γ inhibits Th2 cell polarization [80]. These distinct Th cell subsets alter the behavior of target cells and tailor the immune response depending on the microenvironmental cues they receive by secreting unique repertoires of cytokines that both mediate their responses and provide helper functions to other immune cells [90]. Th1 cells produce IL-2 and IFN-γ, which direct CTL responses, whereas Th2 cells produce IL-4, IL-10, and IL-13 and facilitate local humoral immune (HI) responses [91]. Th1 cytokines also promote the polarization of macrophages toward the tumoricidal M1 phenotype, whereas Th2 cytokines induce T cell anergy and enhance the polarization of macrophages toward the tumor-promoting M2 phenotype [88].

Notably, a single neonatal administration of BPA (250 μg/kg bw) increased subsequent tumor growth when 4T1 breast cancer cells were inoculated in the mammary fat pad of female BALB/c mice upon sexual maturity [64]. This increased tumor growth was related to a larger proportion of forkhead box P3-positive (FOXBP+) Treg cells in infiltrates of tumors from BPA-exposed mice compared to tumors from mice not exposed to BPA [64]. In the same study, tumors from BPA-exposed mice also had more arginase expression (indication of M2 macrophage) and less pro-inflammatory cytokine expression (e.g., IFN-γ and tumor necrosis factor alpha [TNF-α]), suggesting that BPA may contribute to the formation of an unfavorable immune microenvironment for destroying cancer cells. Importantly, the authors addressed that the local or systemic environment, rather than cancer cells, might mediate the pro-tumorigenic effect of BPA. However, the study did not explore whether certain immune cell types (e.g., Treg cells and M macrophages) directly cause this unfavorable immune microenvironment or if it is a result of more aggressive tumors induced by other stromal factors rather than immune cells (e.g., fibroblasts and adipocytes), as described in Section 3 and Section 4, respectively. It should also be noted that BPA was administered subcutaneously rather than by the typical route (oral exposure), so further work is needed to validate these results using a common route of exposure (chronic- and low-dose) to administer BPA and, besides, elucidate the mechanisms of BPA-induced immune cell subset drift toward the anti-inflammatory phenotype in the TME. A more recent study conducted by the same group reported results conflicting with their previous findings [64]; the same BPA exposure and tumor-establishment protocol induced a pro-inflammatory intratumoral microenvironment (i.e., increased protein expression of IL-1β, IL-6, IFN-γ, and TNF-α) at the metastatic stage, that was related to increased metastasis of breast cancer cells to the lung [92]. Thus, whether BPA exposure leads to a pro- or anti-inflammatory condition may also be complicated by the cancer development stage.

Although the effect of BPA on Th cell polarization is rarely studied in the context of cancer, the enhancement of Th2 polarization and function has been well-studied as a mechanism that contributes to allergic diseases induced by BPA exposure [60,61]. For example, BPA administered intratracheally enhanced ovalbumin (OVA)-induced lung inflammation [60]. Specifically, intratracheal exposure to BPA (weekly for 6 weeks) in the presence of OVA in juvenile mice increased the level of Th2-promoting cytokines and chemokines (e.g., IL-33 and CCL5) in the lungs, as well as circulating OVA-specific immunoglobulin E, indicating Th2 activation. Moreover, low and medium doses of BPA (0.0625 and 1.25 pM/mice/week) enhanced allergic inflammation more potently than a higher dose of BPA (25 pM/mice/week) [60]. Additionally, treatment with BPA (3 μM) increased the IL-4 production by mesenteric lymph node cells derived from mice exposed to *Trichinella spiralis* infection, an infection reported to induce a strong Th2 immune response [62]. The same study further demonstrated differential BPA effects on Th1 and Th2 responses using strains of mice (e.g., BALB/c and C57BL/6) differentially susceptible to the parasite *Leishmania major*. Susceptibility to *L. major* in BALB/c mice has been attributed to a defective Th1 immune response and a predominant Th2 response, whereas resistant C57BL/6 mice develop a primarily Th1 immune response [62]. BPA treatment (3 or 10 μM) increased IL-4 production in splenocytes isolated from *L. major*-infected BALB/c mice, whereas it did not affect the INF-γ production in splenocytes isolated from *L. major*-infected C57BL/6 mice. Therefore, BPA exposure may increase the Th2 response by increasing IL-4 production without affecting the Th1 response [62]. Moreover, BPA can preferentially polarize Th2 cells in CD14+ monocyte-derived DCs and allogeneic naïve T cells (CD4+/CD45RO-) isolated from healthy humans [63]. DCs are efficient APCs capable of initiating, coordinating, and regulating adaptive immune responses. During antigen presentation, DCs upregulate the synthesis of co-stimulatory receptor molecules (e.g., CD86, CD80, CD83, and CD40) on their surface [93]. These activated DC receptor molecules bind their co-stimulatory molecules on Th cells, which triggers DCs to secrete IL-12 or IL-10, and thereby ultimately determines Th1/Th2 differentiation [94]. The production of IL-10 relative to IL-12 increased after pretreatment with BPA in the presence of TNF-α from DCs upon CD40 ligation using CD40 ligand-transfected cells, which mimics the engagement of T cells to induce cytokine production by DCs [63]. When DCs were treated with BPA in the presence of TNF-α, then subsequently cultured with allogenic naïve Th cells, Th2 cytokine levels were increased, but IFN-γ levels did not alter, thereby resulting in a dose-dependent (0.001 to 0.1 nM) increase in IL-5/IFN-γ, IL-10/IFN-γ, and IL-13/IFN-γ. This Th2 deviation induced by BPA/TNF-α-matured DCs was markedly abrogated when the DCs matured in the presence of ICI 182,780, an ER antagonist, suggesting that ERs are involved [63].

Some other studies also suggest that BPA inhibits Th1 polarization or the Th1 response. IFN-γ induced tryptophan degradation and neopterin production in human macrophages, processes mediated by the enzymes indoleamine-2,3-dioxygenase (IDO-1) and guanosine triphosphate cyclohydrolase, respectively [95,96]. Stimulation with PHA caused the same outcomes in PBMCs derived from healthy human subjects [53]. However, BPA treatment (25–200 μM) suppressed IFN-γ production, IDO-1 activity, tryptophan degradation, and neopterin production in PHA-stimulated human PBMCs [53]. One week of BPA pretreatment (2.5 μg/kg bw/day) reduced IFN-γ production in splenic mononuclear cells of both normal C57BL/6 and lupus-prone NZB/NZW mice upon stimulation with concanavalin A in vitro [59]. Notably, the administration of BPA delayed disease onset in NZB/NZW mice, which was accompanied by a substantial reduction in the production of immunoglobin G2a (IgG2a) antibody, probably due to less production of IFN-γ that induces immunoglobulin class switching into IgG2a [59]. Therefore, BPA may enhance Th2 immune responses but suppress Th1 immune responses (Table 1). During Th1 type immune responses, activated T cells release large amounts of cytokines, such as IL-2 and IFN-γ, which induce antimicrobial and anti-tumoral host defense. Th cells exert an anti-tumorigenic effect via their secretion of pro-inflammatory cytokines, and by driving polarization of macrophages toward M1 types and inducing CTL functions [88,90]. Thus, suppressing the Th1 type immune response during BPA exposure may increase the risk of infections and carcinogenesis. However, whether BPA affects Th cell migration in addition to polarization in the context of cancer is largely unknown, and elucidating the effect of BPA on Th cells will be crucial to understanding the pro-tumorigenic effect of BPA.

#### 2.2.2. Infiltration and Polarization of Macrophages

The TAM is the most abundant type of immune cell in the TME. In response to environmental signals, macrophages can polarize toward different phenotypes that display quite different functions; M1 macrophages induce inflammation responses against invading pathogens and cancer cells, whereas immunosuppressive M2 macrophages promote tumor progression as described in Section 2.2. The M1 subtype has high expression of the membrane marker CD11c and secretes inducible nitric oxide synthase (iNOS or NOS2), besides pro-inflammatory cytokines, such as IL-6, monocyte chemoattractant protein (MCP)-1, and TNF-α [78]. By contrast, the M2 subtype highly expresses the surface marker CD206 and produces a high level of arginase (ARG) 1 and anti-inflammatory cytokines, such as transforming growth factor beta (TGF-β) and IL-10 [83]. Exposure to BPA may alter the infiltrating macrophage subset population or macrophage phenotype within the TME, thus changing the macrophage phagocytic function and inflammatory response.

A study suggested that the pro-tumorigenic effect of BPA may be related to an increase in M2 macrophages in the TME. Ductal carcinoma in situ (DCIS) is a non-invasive breast cancer but has a high chance of progressing into invasive breast cancer [97]. Chronic exposure to BPA (2.5 μg/L in drinking water for 70 days,30 days before and 40 days after inoculation) increased the growth and metastasis (into nymph nodes) of tumors derived from DCIS cells xenografted into the mammary fat pads of mice [70]. Notably, the tumors derived from the BPA-exposed mice contained a high level of CD206+ M2 macrophages [70]. Additionally, the same study showed that migration of macrophages (RAW 264.7 cells) was increased under co-culture with DCIS cells, and the migration was more enhanced in the presence of 0.01 μM BPA. In those co-culture conditions, BPA induced macrophage polarization toward a mixed M1/M2 phenotype showing increased expression of both M1 (e.g., NOS2) and M2 (e.g., CD206 and ARG1) markers [70]. However, the study did not identify the phenotypes of the macrophages that migrated. BPA also increased both the proliferation and migration of DCIS cells [70], suggesting that BPA-induced tumor growth may indirectly alter the reciprocal interaction of macrophages with cancer cells and guide the effect of BPA on macrophage behavior. It is thus unclear whether the increase in M2 macrophages in BPA-exposed tumors is due to an increase in migration capacity, polarization, or proliferation and whether this change is induced directly by BPA or indirectly due to an increase in tumor growth. In another study, treatment with BPA and other xenoestrogens (e.g., bis (2-ethylhexyl) phthalate, dibutyl phthalate) at 1 μM increased migration of human PBMC-derived M2 but not M1 macrophages [71], suggesting that BPA exposure may selectively increase the migration of M2 macrophages. M1 and M2 macrophages had markedly different migration capacities toward chemoattractants (e.g., CCL2, CCL5, C-X-C motif chemokine ligand (CXCL) 10, CXCL12, and complement component 1q) that are relevant in neuroinflammatory diseases, such as multiple sclerosis; only M2 macrophages had increased migratory properties [98].

In addition to the change in the infiltration rate of specific immune cell types, phenotypic changes in tumor-infiltrating cells shape the TME. Likewise, the recruitment of specific types of macrophages from circulation can be altered by BPA exposure, and BPA may also increase the population of certain macrophage subtypes by facilitating polarization or proliferation. However, in vitro studies have reported inconsistent results regarding the effect of BPA on M1 or M2 phenotype polarization. BPA promoted or suppressed M1 macrophage polarization or activity depending on experimental conditions, such as the BPA concentration, the macrophage source, or the stimulant used. BPA treatment (4–45 μM) decreased nitric oxide (NO) production and NOS2 protein expression in RAW 264.7 macrophages [65]. The same conditions also suppressed the activation of mitogen-activated protein kinases (MAPKs), besides nuclear factor kappa-light-chain-enhancer of activated B cells (NF-κB), and the nod-like receptor protein 3 activity. Concomitantly, treatment with BPA decreased the expression of pro-inflammatory cytokines, including TNF-α, IL-6, IL-1β, and IL-18, at both the mRNA and protein levels [65]. Similar results were also reported in another study, but BPA-altered macrophage responses only in the presence of LPS; although BPA exposure (1–50 μM) alone did not affect NO and TNF-α production in both mouse peritoneal macrophages and RAW 264.7 cells, it dose-dependently inhibited LPS-induced NO and TNF-α production, as well as TNF--α and NOS2 transcription, processes mediated by ERs [66]. In addition, BPA treatment also reduced the LPS-induced binding activity of NF-κB [66]. Along the same lines, less TNF-α and NO were produced in peritoneal macrophages obtained from BPA-exposed mice (500 mg/kg bw/day injections for 4 weeks) and cultured ex vivo with LPS [67]. A similar effect was observed in peritoneal macrophages isolated from BPA-unexposed mice upon stimulation with LPS in the presence of BPA (10 and 100 μΜ) [67].

Contrary to these studies, others have shown that BPA treatment promotes M1 polarization. Treating mouse peritoneal macrophages with BPA (0.1, 1, and 10 μM) increased the number of M1 macrophages (CD11c+ cells), the transcription level of NOS2 and CD11c, and the secretion of NOS2, in addition to increasing both the expression and secretion of cytokines associated with M1 subtype (e.g., TNF-α, IL-6, and MCP-1) under M1 polarization conditions [68]. Up-regulation of the transcription factor, interferon regulatory factor 5, was suggested to be related to the BPA-induced increase in M1 polarization under the M1 polarization conditions [68]. The same study also reported opposite effects of BPA under M2 polarization conditions: a decrease in the number of M2 macrophages (CD206+ cells), the expression of CD206 and ARG1 transcripts, and the expression and secretion of anti-inflammatory cytokines (e.g., IL-10 and TGF-β). BPA (0.1 μM) also increased IL-6 secretion, TNF-α secretion, and NF-κB activity in a human macrophage cell line (THP1) as well as monocytes isolated by CD14+ selection from human PBMCs [69]. The BPA-induced enhancement of M1 polarization and activity was suppressed by ICI 182,780, suggesting the effects of BPA are mediated by ERs [69]. Another study that further explored the mechanism of NF-κB activation by BPA suggested that BPA is a potential agonist of the TLR4/myeloid differentiation factor 2 complex [99]. According to that study, BPA stimulated downstream adapter molecules, such as myeloid differentiation primary response 88 and IL-1 receptor-associated kinase 4, that ultimately activate NF-κB [99].

Overall, research on the effect of BPA on macrophage polarization has produced inconsistent findings (Table 1). These discrepancies may be partly due to the complexity of macrophage phenotypes. Several subclasses of macrophages have been suggested, although the M1 and M2 classifications have been commonly used [100,101]. At least four distinct subpopulations of macrophages have been characterized through gene-profiling different macrophage populations using multiple surface markers; each subpopulation has a distinct function within the TME in an orthotopic model of lung cancer, where murine lung cancer cells are directly implanted into the left lobe of syngeneic mice [102]. Additionally, within the breast TME, the complex interaction between cancer cells, macrophages, and adaptive immune cells may affect the polarization of macrophages. For example, Th cells play an important role in determining macrophage polarization [88]. The induction of Th2 cell polarization by CCL5 has been shown to promote metastasis in the MMTV-polyoma middle tumor-antigen (PyMT) transgenic mouse model of ER+ breast cancer [83]. In this model, TAMs derived from PyMT mice bearing homozygous null CCL5 (CCL5^−/−^) exhibited a cancer-suppressing phenotype compared to TAMs in control (CCL5^+/+^) PyMT mice. The effect of CCL5 was diminished after inhibiting CD4+ T cells, suggesting that Th cells mediate CCL5-induced cancer-promoting phenotypes of TAMs. It is thus important to consider the cellular interactions in breast TME to adequately assess the effect of BPA on macrophage polarization [78]. Patient-derived explant (PDE) has been proposed to model an intact human mammary tissue microenvironment and cellular interactions of TMEs [85,103]. In a human benign breast PDE culture system, it has been shown that macrophage polarization (i.e., M1 and M2 marker expression) within human tissue is influenced by external cytokines that polarize M1 and M2 macrophages and is affected by treatment with benzophenone-3, an EDC [103]. Moreover, because almost all tissues, including breast tissue, contain macrophages (resident macrophages), altering macrophage polarization using environmental factors could be an important mechanism for providing functional plasticity to macrophages in complex bidirectional interactions with other TME components [104]. Therefore, it is crucial to study the effect of BPA on macrophages in the complexity of the TME to understand better the role of macrophages in mediating BPA-induced cancer-promoting effects.

## 3. Fibroblasts and ECMs

Mammographically dense breast tissue is a well-recognized risk factor for breast cancer [105,106], and breast tumors are often found in dense areas in mammary tissue [107]. Dense breast tissue is associated with increased cellularity, besides excessive deposition of collagen and other ECM components [106]. A recent study further suggested a potential mechanism whereby dense breast tissue induces cancer progression; stiff ECMs inhibited the expression of tumor suppressor miR-203, which led to a concomitant elevation of its target oncogene zinc-finger protein 217 and activated protein kinase B to induce epithelial cell proliferation and motility [108]. Fibroblasts, the most abundant cell type in the mammary stroma, are a key cell type responsible for the synthesis and turnover of ECMs [109,110]. The role of fibroblasts in cancer development and progression has been recognized in various cancers, including breast cancer [111,112,113]. Fibroblasts promote cancer growth, survival, invasion, and migration by secreting growth factors and depositing ECMs [32,114].

BPA exposure may increase collagen deposition and thereby enhance mammary gland stiffness. Serum BPA levels were associated with high mammographic breast density, which was independent of body mass index (BMI) and other covariates in postmenopausal women [115]. Notably, BPA exposure (25 μg/kg bw, daily oral gavage) during pregnancy deregulated genes associated with ECM composition (e.g., type I collagen) in the fibroblasts of female mouse offspring [116]. This gene dysregulation was related to increased collagen deposition and gland stiffness at 12 weeks of age when ductal elongation is completed [116]. Additionally, administration of BPA (5, 20, and 100 mg/kg bw/day) via oral gavage dose-dependently induced cardiac fibrosis in rats [117]. In line with this, BPA treatment (0.1–10 μM) increased the proliferation and collagen production of cardiac fibroblasts isolated from 1-to-3-day old neonatal rats [117]. This increased collagen production and cell proliferation in BPA-treated cardiac fibroblasts were mediated via ERs and activation of ERK1/2 [117]. In skin fibroblasts isolated from mice, treatment with BPA (0.1 μM) increased α-smooth muscle actin (SMA) expression and collagen deposition [118]. Moreover, selective inhibition of ERβ using small interfering RNA reversed the effect of BPA on α-SMA expression and collagen deposition [118].

In addition to collagen synthesis, the ECM turnover rate determines ECM degradation and accumulation. ECM turnover is largely regulated by the balance of metalloproteinases (MMPs) and tissue inhibitors of metalloproteinases (TIMPs) [119]. A relatively high dose of BPA treatment (10 μM) changed the MMPs/TIMPs balance in human trophoblastic Jeg-3 cells by decreasing the mRNA levels of MMP2 and MMP9 but increasing TIMP1 [120]. In that study, the TIMP level induced by BPA was reversed in the presence of ICI 182,780 but not G15 (a high-affinity and selective GPER antagonist), suggesting ERs rather than GPER mediate TIMP overproduction. These studies suggest that fibroblasts are a crucial target of BPA that leads to collagen accumulation and that ERs mediate BPA regulation of collagen synthesis and degradation.

Distinct roles of ERα and ERβ in the regulation of collagen synthesis and degradation were demonstrated using ERα^−/−^ and ERβ^−/−^ mice [121]. Collagen content was increased in the skin of ERα^−/−^ mice but decreased in the skin of ERβ^−/−^ mice [121]. Contradictorily, collagen production was enhanced in fibroblasts derived from ERβ^−/−^ mice [121]. However, these fibroblasts also exhibited increased levels of MMPs (e.g., MMP-8 and MMP-15) and decreased expression of small leucine-rich proteoglycans, such as lumican and decorin, which are associated with collagen fibril formation [122]; by contrast, the MMP-15 level was decreased in skin fibroblasts derived from ERα^−/−^ mice [121]. Therefore, ERβ may have a role in the inhibition of collagen degradation and an increase in collagen fibrillogenesis.

A study reported a hormone receptor expression change in female Mongolian gerbils upon exposure to BPA during pregnancy and lactation [123]. The protein expression level of ERα was increased, but that of ERβ markedly decreased in the epithelial cells of mammary glands exposed to BPA compared to mammary glands exposed to the vehicle or water [123]. However, the study did not find any alteration in either ERα or ERβ expression in stromal cells after BPA exposure. Notably, the same study reported elevated protein expression of enhancer of zeste homolog 2 (EZH2) in BPA-exposed mammary glands [123]. EZH2 is a histone methyltransferase that catalyzes tri-methylation of histone H3 at Lys 27 (H3K27me3) to ultimately repress transcription. Dysregulation of EZH2 has been implicated in the development and progression of various cancers, including breast cancer [124]. These results indicate possible epigenetic modification by BPA treatment in mammary glands, but the involvement of an epigenetic mechanism was not investigated in relation to BPA-induced ER expression changes [123]. Prenatal exposure to BPA resulted in gene expression changes in collagen genes in adult fibroblasts [116]. Early developmental epigenetic modification is believed to alter later life phenotypes. In this context, BPA exposure during a period of increased sensitivity to the regulatory effects of an epigenetic mechanism may lead to collagen deposition. It is thus imperative to understand the epigenetic alterations in genes encoding collagens and ERs in humans. Considering that epigenetic changes are potentially reversible, it is also important to elucidate if adverse epigenetic programming by early-life exposure to BPA can be reversed later in adulthood by intervention.

**Figure 1:**
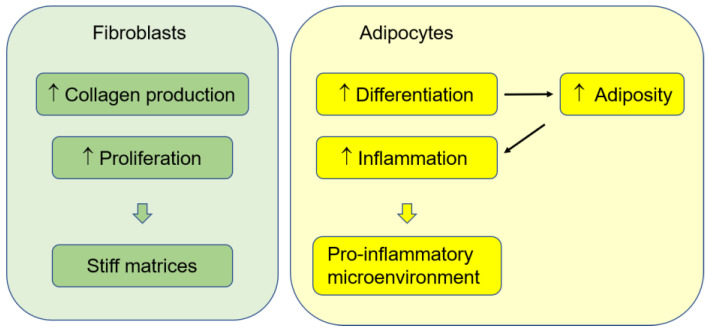
Effects of bisphenol A (BPA) on fibroblasts and adipocytes in the breast tumor microenvironment. BPA increases the proliferation of fibroblasts and induces collagen production in fibroblasts. BPA also increases adiposity due to epigenetic modifications in critical genes (e.g., mesoderm-specific transcript, peroxisome proliferator-activated receptor-gamma) and induces adipocyte inflammation in adipocytes dependently and independently of adiposity.

Additionally, BPA treatment (1 μM) increased the proliferation of breast tumor-derived fibroblasts, in addition to SKBR3 breast cancer cells, and the conditioned medium from the BPA-treated fibroblasts enhanced the migration of SKBR3 cells in a GPER-dependent manner [26], suggesting that BPA may also affect the secretion of factors that promote cancer cell migration in fibroblasts. BPA (0.1 and 1 μM) was also shown to induce proliferative effects in both breast cancer cell lines (SKBR3 and MDA-MB-231 cells) and vascular endothelial cells under hypoxic conditions, which was associated with the GPER-mediated increase in hypoxia-inducible factor 1-α and vascular endothelial growth factor expression [125]. The same study demonstrated that BPA treatment induced a pro-angiogenic effect, increasing migration and tube formation in endothelial cells under in vitro hypoxic conditions and enhancing angiogenesis (CD34 staining) in MDA-MB-231 cell-xenografts established in mouse mammary fat pads. Therefore, BPA exposure may increase the proliferation of stromal cells and predispose these cells to increase the production of the ECM (particularly during the critical time in mammary development), altering tissue architecture and increasing the development of cancer (Figure 1). However, whether this change in stromal cells conclusively enhances breast cancer development or progression needs to be further evaluated.

## 4. Adipocytes

Obesity has been shown to increase the risk of various cancers, including breast cancer [126]. A meta-analysis revealed that obesity is a risk factor for hormone receptor-positive breast cancer in postmenopausal women; obese women with a high BMI (≥30 kg/m^2^) had a higher risk (approximately 40%) than women with a normal BMI (<25 kg/m^2^) [127]. After menopause, peripheral aromatization of androgen precursors is mainly responsible for estrogen biosynthesis. This increased aromatase expression in the adipose tissue has been speculated to increase the risk of breast cancer in obese, postmenopausal women [128,129]. Additionally, obesity is linked with metabolic alterations and chronic inflammation, which increase the propensity to form cancer [130]. The positive energy balance in the obese state increases the circulation of pro-tumorigenic adipokines (e.g., leptin), growth factors (e.g., insulin-like growth factor), and inflammatory cytokines (e.g., IL-6), which can chronically activate metabolic signaling pathways that may potentiate cancer growth [130,131,132].

**Figure 2:**
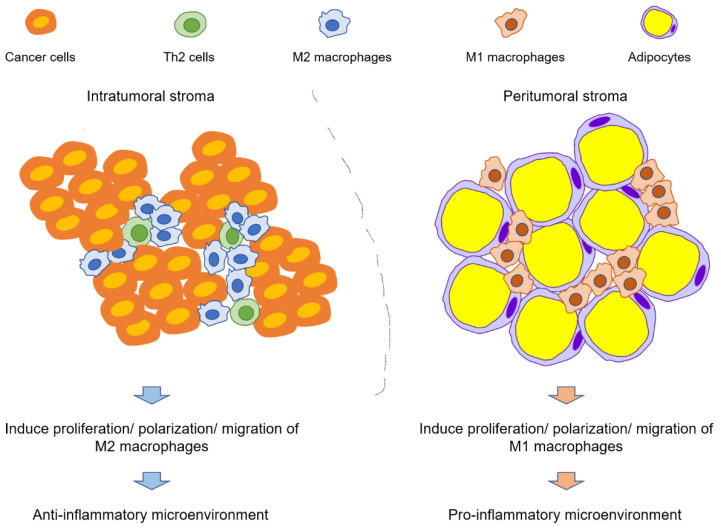
Differential macrophage phenotypes induced by exposure to bisphenol A (BPA) depending on location. BPA induces an anti-inflammatory immune microenvironment (inducing M2 phenotypes) in intratumor tissue (composed of Th cells, fibroblasts, and macrophages) and a pro-inflammatory immune microenvironment (inducing M1 phenotypes) in peritumor tissue (composed of adipocytes, fibroblasts, and macrophages).

Moreover, adipose tissues are the main component of the breast, and dysfunctional adipocytes can significantly impact breast cancer development and progression [133,134,135]. Obesity increases inflammation in mammary glands in both mice and humans [134,136], causing pro-tumorigenic effects [137]. In particular, the crown-like structures of breast adipose tissue (CLS-B, the clustering of macrophages to surround a dead/dying adipocyte) are attributed to inflammatory changes that promote cancer progression in mammary tissues [138]. Obese women who develop breast cancer have a worsened prognosis than non-obese women with breast cancer [139], and the number of CLS-B was related to poor prognosis [140]. Another study further suggested that breast adipose tissue macrophages (BATMs) had a stronger correlation with breast cancer survival than breast tumor stroma macrophages, although there was a strong correlation between both, and that BATMs were negatively associated with both disease-free survival and overall survival [141]. Notably, macrophages found in CLS-B are often M1 [136], indicating that the pro-tumorigenic effect of macrophages is mediated by the M1 phenotype in adipose tissue adjacent to the tumor rather than the intratumoral M2 phenotype (Figure 2).

In both epidemiologic and experimental studies, exposure to BPA has been shown to increase the risk of developing obesity [142,143]. Continuous exposure to low-dose BPA (0.01–1 μM for 14–21 days) enhanced adipogenic differentiation of both human adipose-derived stem cells and mesenchymal stem cells through an increase in gene expression related to adipogenesis (e.g., PPAR-γ, CCCAAT-enhancer-binding protein alpha (C/EBP-α), and lipoprotein lipase) via ER activation [144,145]. Studies have also suggested that prenatal and early postnatal epigenetic modifications related to BPA exposure may predispose individuals and their offspring to promoted adipocyte differentiation [146,147]. Male newborn rats exposed to maternal BPA (5 mg/L in drinking water, 2 weeks before mating and during pregnancy and lactation) had markedly increased postnatal fat mass, accompanying increased protein expression of important genes in adipogenesis and lipogenesis (PPAR-γ, CEBP-α, and sterol regulatory element-binding protein 1) in adipose tissues [148]. The epigenetic modification was suggested as a mechanism underlying BPA-induced enhanced adipogenesis because treatment of BPA (1–20 μM for 5 days) to preadipocytes derived from 1-day-old rat dose-dependently increased the protein expression of DNA-methyltransferase 3A (DNMT3A), but the effect of BPA exposure on DNA methylation in specific genes was not investigated [148]. DNMT3A catalyzes de novo DNA methylation and is thus critical for genetic imprinting [149]. In line with the involvement of epigenetic change in BPA exposure-induced obesity, BPA (1 nM over 8 days) was shown to promote epigenetic changes at the promoter of PPAR-γ, a master regulator of adipogenesis, in 3T3-L1 preadipocytes [150]. In that study, BPA induced a decrease in DNA methylation in PPAR-γ promoter and correspondingly increased the mRNA expression of PPAR-γ [150]. However, the BPA-induced epigenetic change was transient; it only occurred 4 days after starting differentiation. DNA methylation was investigated only in PPAR-γ in that study, and thus, epigenetic regulation in other genes may be required to better relate the epigenetic mechanism with BPA-induced adipogenicity. Studies on a genome-wide analysis of epigenetic changes induced by BPA exposure are rare. One study compared BPA levels in the urine of mothers during pregnancy and genome-wide DNA methylation in the cord blood of their children [146]. It was found that prenatal BPA exposure was related to hypo-methylated CpG in the promoter of obesity-related gene. mesoderm-specific transcript (MEST) and higher BMI *z*-scores (measures of relative weight adjusted for child age and sex) at the age of 6 years [146]. The same study also experimentally demonstrated that mice offspring of BPA-exposed mothers (1 week before mating until delivery) exhibited hypo-methylation of the MEST promoter region in their adipose tissues and developed markedly higher body weight and fat mass at 10 weeks after delivery compared to unexposed animals. Hypo-methylation of the MEST promoter region and the corresponding enhancement of MEST expression were also observed upon BPA exposure during adipocyte differentiation in human adipose-derived mesenchymal stem cells [146]. This evidence provides a mechanistic explanation for how prenatal BPA exposure potentially leads to overweight development in the offspring. During early mammalian development, the genome undergoes dynamic changes in DNA methylation, and thus epigenetic modification is a plausible mechanism for BPA-induced adverse effects, including obesity. The major epigenetic mechanisms include DNA methylation, histone modification, and various RNA-mediated processes, including non-coding RNAs [151]. Simultaneous studies on these different levels of epigenetic modification would provide better insight into their role in BPA-induced adverse effects.

Excess fat accumulation is usually accompanied by chronic inflammation [152], which means that BPA might enhance inflammation and insulin resistance by inducing adiposity. Oral administration of low-dose BPA (as low as 5 μg/kg bw/day) increased body weight, fat mass, circulating inflammatory cytokines, and local inflammation in white adipose tissues in both male and female C57BL/6J mice fed a normal diet [153]. Additionally, the subjects with higher plasma BPA levels had higher waist circumference (visceral adiposity), insulin resistance, and levels of both triglycerides and inflammatory molecules (e.g., IL-6) [154]. However, BPA may also induce inflammatory signaling in adipocytes and increase glucose tolerance independent of obesity induction. In mice fed a high-fat diet, long-term oral administration of BPA (50 μg/kg bw/day, 12 weeks) induced increased glucose intolerance without altering body weight or the percentage of body fat [155]. Moreover, in cultured adipocytes derived from human subcutaneous tissue and 3T3-L1 preadipocytes, treatment with 1 nM BPA did not enhance adipocyte differentiation but reduced insulin sensitivity and glucose utilization and increased the release of pro-inflammatory molecules (e.g., IL-6 and IFN-γ) in accordance with increasing the activation of c-Jun N-terminal kinases as well as signal transducer and activator of transcription 3 [156]. Treatment with BPA (0.1 nM) also GPER-dependently increased the transcription of IL-6, IL-8, and MCP-1 in mammary adipocytes obtained from overweight female patients undergoing surgical mammary reduction [157]. Such results show that BPA can contribute to the pro-inflammatory and pro-tumorigenic microenvironment by inducing adiposity through epigenetic modification of critical genes related to adipogenesis and increasing inflammation by indirectly enhancing adiposity or directly inducing inflammatory signaling in adipose tissue (Figure 1).

BPA accumulates in adipose tissue due to its lipophilic nature, leading to continuous local exposure. In turn, the effects of BPA in adipose tissue can significantly contribute to the formation of a permissive TME. However, future studies are needed to elucidate how BPA exposure affects CLS-B formation and whether BPA-induced inflammation in adipose tissue provides an additional cancer-promoting effect compared to adiposity alone. Additionally, the pro-tumorigenic effect of macrophages is mediated by intratumoral M2 phenotypes and peritumoral (i.e., the tissue surrounding the tumor) M1 phenotypes. If BPA administration induces an anti-inflammatory microenvironment within the tumor but a pro-inflammatory microenvironment in adipose tissue simultaneously; the mechanism of these opposite effects must be explicated. One possibility is that BPA may alter the phenotypes of fibroblasts, adipocytes, and possibly Th cells, leading to pro- or anti-inflammatory microenvironments depending on location (i.e., intratumoral or peritumoral; Figure 2). Different microenvironments may direct the polarization of macrophages into pro- or anti-inflammatory phenotypes, and the interactions of macrophages with other cells may cumulatively influence cancer progression. Moreover, the cancer stage may also affect the BPA-induced pro- or anti-inflammatory microenvironment.

## 5. Conclusions

Not only is BPA ubiquitous, but it can also accumulate in the body, leading to continuous BPA exposure. Exposure to BPA has been thought to promote the growth of ER+ breast cancer cells due to its ability to mimic estrogen. BPA may also significantly contribute to tumor development and progression by facilitating the formation of a permissive TME. BPA can affect various cell types and ECMs that compose TMEs; it may reduce the proliferation of CTLs (most likely by shortening telomerase length), increase deposition of ECMs, and enhance inflammation in mammary adipose tissue. BPA alters immune cells, fibroblasts, and adipocytes via ERs, GPER, or both receptors. Although it is evident that BPA can alter TME to favor cancer formation and growth, it is less clear whether BPA directly influences the migration or polarization of Th cells or macrophages. Moreover, further work is needed to evaluate the impact of the changes in TME components induced by BPA exposure in the context of human health. Future studies that determine whether these changes contribute to more aggressive tumors will greatly enhance the understanding of the role of BPA exposure in cancer development and progression. Knowledge of the role of BPA in pro-tumorigenic microenvironment formation is expanding. Thus, this review did not provide mechanistic details for the continuous and stepwise changes at the level of the stromal components to the formation of cancer and progression to metastatic cancer. Particularly, information on how BPA impacts cancer-stromal interactions is lacking. This review also included limited human studies, and these studies did not assess long-term BPA exposure.

## Figures and Tables

**Table 1:** Functions of immune cells in tumor microenvironment (TME) and potential effects of bisphenol A (BPA) on immune cells.

Cell Type	Subtype	Function in TME	Effect of BPA
CD8+ cells (CTLs)		Eliminate cancer cells	Reduces telomere length [52,54,55] Suppresses proliferation [55]
CD4+ cells (Th cells)	Th1	Produce pro-inflammatory immune microenvironments (secrete IL-2, IFN-γ) Induce CTL response Induce M1 macrophage polarization Eliminate cancer cells	Inhibits polarization/function (inhibits IFN-γ production) in allergen-stimulated conditions [53,59]
	Th2	Produce anti-inflammatory immune microenvironment (secrete IL-4, IL-10) Induce humoral immune response Induce M2 macrophage polarization Increase cancer growth and migration	Enhances polarization/function in allergic disease models [60,61] and in parasite infection [62] Induces polarization by dendritic cells upon maturation with BPA/TNF-α [63]
	Treg	Induce anti-inflammatory immune microenvironment	Derives large proportion in breast tumors grown on BPA-exposed mice [64]
Macrophages	M1	Induce pro-inflammatory microenvironment Eliminate cancer cells	Inhibits polarization in RAW 264.7 macrophages [65,66] and ex vivo culture of mouse peritoneal macrophages [67] Promotes polarization in mouse peritoneal macrophages [68] and THP1 cells [69]
	M2	Induce anti-inflammatory microenvironment Increase tumor promotion	Derives large proportion in breast tumors grown on BPA-exposed mice [64] Derives large content in BPA-exposed before and during cancer formation in mice [70] Induces migration in human PBMCs [71]

CTL: cytotoxic T cell, Th cell: T helper cell, Treg: regulatory T cell, IL-2: interleukin-2, INF-γ: interferon-gamma, IL-4: interleukin-4, IL-10: interleukin-10, TNF-α: tumor necrosis factor-alpha, PBMC: peripheral blood mononuclear cell.
